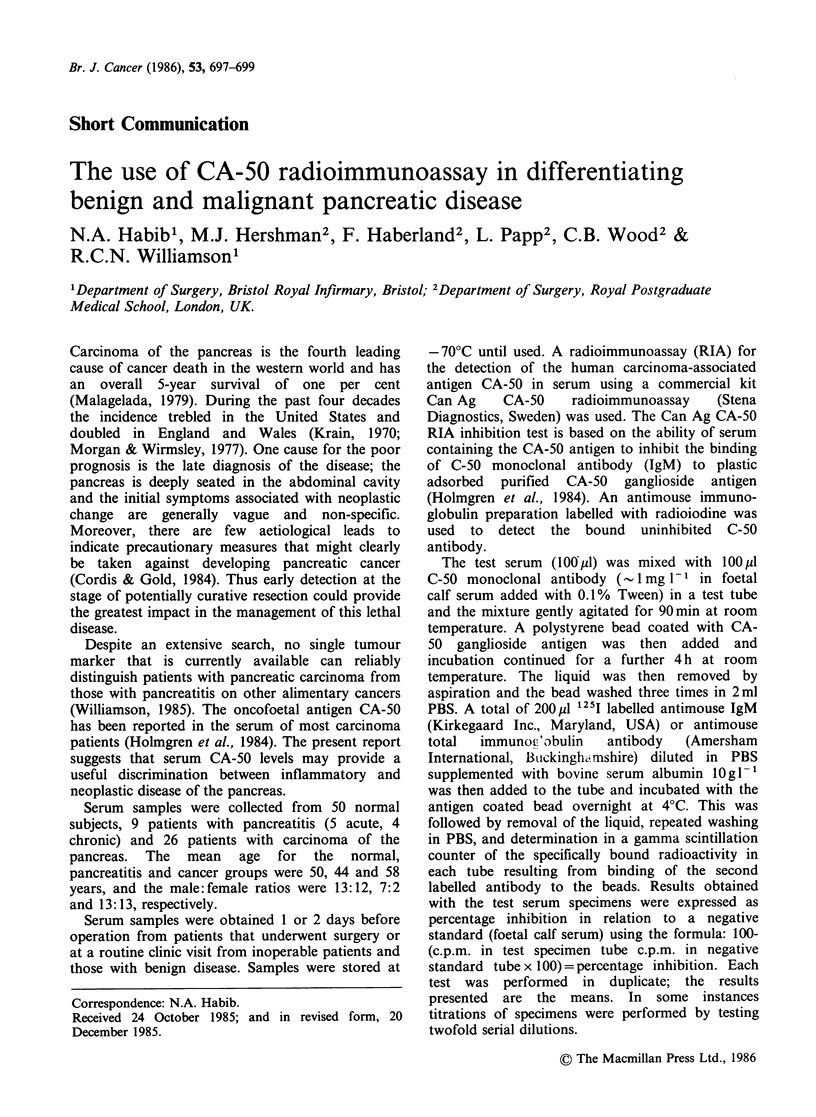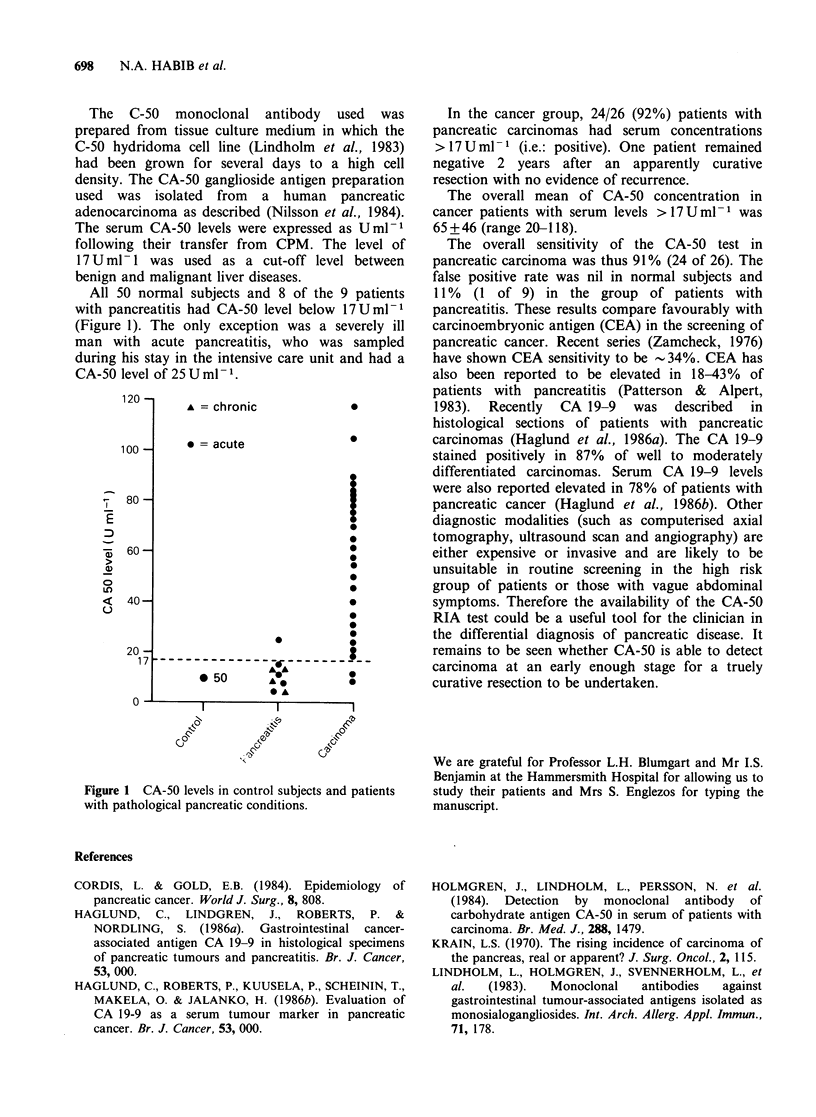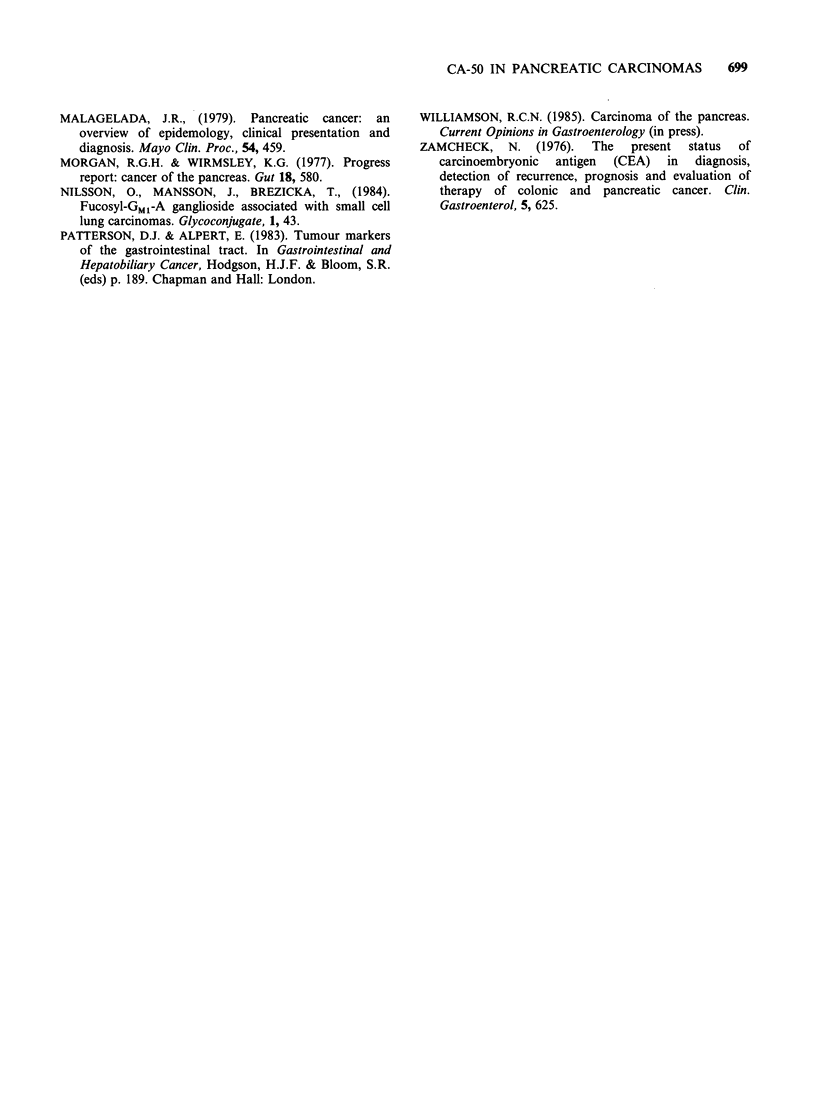# The use of CA-50 radioimmunoassay in differentiating benign and malignant pancreatic disease.

**DOI:** 10.1038/bjc.1986.116

**Published:** 1986-05

**Authors:** N. A. Habib, M. J. Hershman, F. Haberland, L. Papp, C. B. Wood, R. C. Williamson


*Br. J. Cancer* (1986), **53**, 697–699

### Short Communication

### The use of CA-50 radioimmunoassay in differentiating benign and malignant pancreatic disease

N.A. Habib[1], M.J. Hershman[2], F. Haberland[2], L. Papp[2], C.B. Wood[2] & R.C.N. Williamson[1]

[1]*Department of Surgery, Bristol Royal Infirmary, Bristol;* [2]*Department of Surgery, Royal Postgraduate Medical School, London, UK.*

Carcinoma of the pancreas is the fourth leading cause of cancer death in the western world and has an overall 5-year survival of one per cent (Malagelada, 1979). During the past four decades the incidence trebled in the United States and doubled in England and Wales (Krain, 1970; Morgan & Wirmsley, 1977). One cause for the poor prognosis is the late diagnosis of the disease; the pancreas is deeply seated in the abdominal cavity and the initial symptoms associated with neoplastic change are generally vague and non-specific. Moreover, there are few aetiological leads to indicate precautionary measures that might clearly be taken against developing pancreatic cancer (Cordis & Gold, 1984). Thus early detection at the stage of potentially curative resection could provide the greatest impact in the management of this lethal disease.

Despite an extensive search, no single tumour marker that is currently available can reliably distinguish patients with pancreatic carcinoma from those with pancreatitis on other alimentary cancers (Williamson, 1985). The oncofoetal antigen CA-50 has been reported in the serum of most carcinoma patients (Holmgren *et al.*, 1984). The present report suggests that serum CA-50 levels may provide a useful discrimination between inflammatory and neoplastic disease of the pancreas.

Serum samples were collected from 50 normal subjects, 9 patients with pancreatitis (5 acute, 4 chronic) and 26 patients with carcinoma of the pancreas. The mean age for the normal, pancreatitis and cancer groups were 50, 44 and 58 years, and the male:female ratios were 13:12, 7:2 and 13:13, respectively.

Serum samples were obtained 1 or 2 days before operation from patients that underwent surgery or at a routine clinic visit from inoperable patients and those with benign disease. Samples were stored at −70°C until used. A radioimmunoassay (RIA) for the detection of the human carcinoma-associated antigen CA-50 in serum using a commercial kit Can Ag CA-50 radioimmunoassay (Stena Diagnostics, Sweden) was used. The Can Ag CA-50 RIA inhibition test is based on the ability of serum containing the CA-50 antigen to inhibit the binding of C-50 monoclonal antibody (IgM) to plastic adsorbed purified CA-50 ganglioside antigen (Holmgren *et al.*, 1984). An antimouse immunoglobulin preparation labelled with radioiodine was used to detect the bound uninhibited C-50 antibody.

The test serum (100 $\mu$l) was mixed with 100 $\mu$l C-50 monoclonal antibody ($\sim 1\,\mathrm{mg\,l^{-1}}$ in foetal calf serum added with 0.1% Tween) in a test tube and the mixture gently agitated for 90 min at room temperature. A polystyrene bead coated with CA-50 ganglioside antigen was then added and incubation continued for a further 4 h at room temperature. The liquid was then removed by aspiration and the bead washed three times in 2 ml PBS. A total of 200 $\mu$l $^{125}$I labelled antimouse IgM (Kirkegaard Inc., Maryland, USA) or antimouse total immunoglobulin antibody (Amersham International, Buckinghamshire) diluted in PBS supplemented with bovine serum albumin $10\,\mathrm{g\,l^{-1}}$ was then added to the tube and incubated with the antigen coated bead overnight at 4°C. This was followed by removal of the liquid, repeated washing in PBS, and determination in a gamma scintillation counter of the specifically bound radioactivity in each tube resulting from binding of the second labelled antibody to the beads. Results obtained with the test serum specimens were expressed as percentage inhibition in relation to a negative standard (foetal calf serum) using the formula: 100-(c.p.m. in test specimen tube c.p.m. in negative standard tube × 100) = percentage inhibition. Each test was performed in duplicate; the results presented are the means. In some instances titrations of specimens were performed by testing twofold serial dilutions.

Correspondence: N.A. Habib.
Received 24 October 1985; and in revised form, 20 December 1985.

© The Macmillan Press Ltd., 1986

The C-50 monoclonal antibody used was prepared from tissue culture medium in which the C-50 hydridoma cell line (Lindholm *et al.*, 1983) had been grown for several days to a high cell density. The CA-50 ganglioside antigen preparation used was isolated from a human pancreatic adenocarcinoma as described (Nilsson *et al.*, 1984). The serum CA-50 levels were expressed as U ml$^{-1}$ following their transfer from CPM. The level of 17 U ml$^{-}$1 was used as a cut-off level between benign and malignant liver diseases.

All 50 normal subjects and 8 of the 9 patients with pancreatitis had CA-50 level below 17 U ml$^{-1}$ (Figure 1). The only exception was a severely ill man with acute pancreatitis, who was sampled during his stay in the intensive care unit and had a CA-50 level of 25 U ml$^{-1}$.

**Figure 1** CA-50 levels in control subjects and patients with pathological pancreatic conditions.

In the cancer group, 24/26 (92%) patients with pancreatic carcinomas had serum concentrations $>17$ U ml$^{-1}$ (i.e.: positive). One patient remained negative 2 years after an apparently curative resection with no evidence of recurrence.

The overall mean of CA-50 concentration in cancer patients with serum levels $>17$ U ml$^{-1}$ was $65 \pm 46$ (range 20–118).

The overall sensitivity of the CA-50 test in pancreatic carcinoma was thus 91% (24 of 26). The false positive rate was nil in normal subjects and 11% (1 of 9) in the group of patients with pancreatitis. These results compare favourably with carcinoembryonic antigen (CEA) in the screening of pancreatic cancer. Recent series (Zamcheck, 1976) have shown CEA sensitivity to be $\sim 34\%$. CEA has also been reported to be elevated in 18–43% of patients with pancreatitis (Patterson & Alpert, 1983). Recently CA 19–9 was described in histological sections of patients with pancreatic carcinomas (Haglund *et al.*, 1986*a*). The CA 19–9 stained positively in 87% of well to moderately differentiated carcinomas. Serum CA 19–9 levels were also reported elevated in 78% of patients with pancreatic cancer (Haglund *et al.*, 1986*b*). Other diagnostic modalities (such as computerised axial tomography, ultrasound scan and angiography) are either expensive or invasive and are likely to be unsuitable in routine screening in the high risk group of patients or those with vague abdominal symptoms. Therefore the availability of the CA-50 RIA test could be a useful tool for the clinician in the differential diagnosis of pancreatic disease. It remains to be seen whether CA-50 is able to detect carcinoma at an early enough stage for a truely curative resection to be undertaken.

We are grateful for Professor L.H. Blumgart and Mr I.S. Benjamin at the Hammersmith Hospital for allowing us to study their patients and Mrs S. Englezos for typing the manuscript.

### References

CORDIS, L. & GOLD, E.B. (1984). Epidemiology of pancreatic cancer. *World J. Surg.*, **8**, 808.

HAGLUND, C., LINDGREN, J., ROBERTS, P. & NORDLING, S. (1986*a*). Gastrointestinal cancer-associated antigen CA 19–9 in histological specimens of pancreatic tumours and pancreatitis. *Br. J. Cancer*, **53**, 000.

HAGLUND, C., ROBERTS, P., KUUSELA, P., SCHEININ, T., MAKELA, O. & JALANKO, H. (1986*b*). Evaluation of CA 19-9 as a serum tumour marker in pancreatic cancer. *Br. J. Cancer*, **53**, 000.

HOLMGREN, J., LINDHOLM, L., PERSSON, N. *et al.* (1984). Detection by monoclonal antibody of carbohydrate antigen CA-50 in serum of patients with carcinoma. *Br. Med. J.*, **288**, 1479.

KRAIN, L.S. (1970). The rising incidence of carcinoma of the pancreas, real or apparent? *J. Surg. Oncol.*, **2**, 115.

LINDHOLM, L., HOLMGREN, J., SVENNERHOLM, L., *et al.* (1983). Monoclonal antibodies against gastrointestinal tumour-associated antigens isolated as monosialogangliosides. *Int. Arch. Allerg. Appl. Immun.*, **71**, 178.

MALAGELADA, J.R., (1979). Pancreatic cancer: an overview of epidemology, clinical presentation and diagnosis. *Mayo Clin. Proc.*, **54**, 459.

MORGAN, R.G.H. & WIRMSLEY, K.G. (1977). Progress report: cancer of the pancreas. *Gut* **18**, 580.

NILSSON, O., MANSSON, J., BREZICKA, T., (1984). Fucosyl-$G_{M1}$-A ganglioside associated with small cell lung carcinomas. *Glycoconjugate*, **1**, 43.

PATTERSON, D.J. & ALPERT, E. (1983). Tumour markers of the gastrointestinal tract. In *Gastrointestinal and Hepatobiliary Cancer*, Hodgson, H.J.F. & Bloom, S.R. (eds) p. 189. Chapman and Hall: London.

WILLIAMSON, R.C.N. (1985). Carcinoma of the pancreas. *Current Opinions in Gastroenterology* (in press).

ZAMCHECK, N. (1976). The present status of carcinoembryonic antigen (CEA) in diagnosis, detection of recurrence, prognosis and evaluation of therapy of colonic and pancreatic cancer. *Clin. Gastroenterol*, **5**, 625.